# Supplementary material for: Comparative genomics provides new insights into the diversity, physiology, and sexuality of the only industrially exploited tremellomycete: Phaffia rhodozyma
Source: BMC Genomics. 2016 Nov 9;17:901. doi: 10.1186/s12864-016-3244-7 (PMC5103461; doi:10.1186/s12864-016-3244-7)
Supplement: Additional file 6: — List of orphan genes with links to PFAM (related to Additional file 1: Table S1). (ZIP 1428 kb) [file 12864_2016_3244_MOESM6_ESM.zip › PFAM_UNIQUE/G04932_P.html]

#                                                                            --- full sequence --- -------------- this domain -------------   hmm coord   ali coord   env coord
  
# target name        accession   tlen query name           accession   qlen   E-value  score  bias   #  of  c-Evalue  i-Evalue  score  bias  from    to  from    to  from    to  acc description of target
  
#------------------- ---------- ----- -------------------- ---------- ----- --------- ------ ----- --- --- --------- --------- ------ ----- ----- ----- ----- ----- ----- ----- ---- ---------------------
  
G04932\_P             -            583 DUF812               PF05667.6    594       6.6    3.8  13.1   1   3       3.9     1e+03   -3.5   0.0   334   371    37    74    27    80 0.75 - Protein of unknown function (DUF812)  
G04932\_P             -            583 DUF812               PF05667.6    594       6.6    3.8  13.1   2   3     0.033       8.5    3.4   1.6   321   397   255   338   242   354 0.71 - Protein of unknown function (DUF812)  
G04932\_P             -            583 DUF812               PF05667.6    594       6.6    3.8  13.1   3   3    0.0042       1.1    6.3   2.8   185   338   411   572   382   576 0.67 - Protein of unknown function (DUF812)
